# Velocity-Aided Attitude Estimation for Helicopter Aircraft Using Microelectromechanical System Inertial-Measurement Units

**DOI:** 10.3390/s16122102

**Published:** 2016-12-11

**Authors:** Sang Cheol Lee, Sung Kyung Hong

**Affiliations:** Department of Aerospace Engineering, Sejong University, Seoul 143-747, Korea; lscjjang14@naver.com

**Keywords:** microelectromechanical system (MEMS), inertial-measurement unit (IMU), airspeed, attitude estimation, complementary filter

## Abstract

This paper presents an algorithm for velocity-aided attitude estimation for helicopter aircraft using a microelectromechanical system inertial-measurement unit. In general, high- performance gyroscopes are used for estimating the attitude of a helicopter, but this type of sensor is very expensive. When designing a cost-effective attitude system, attitude can be estimated by fusing a low cost accelerometer and a gyro, but the disadvantage of this method is its relatively low accuracy. The accelerometer output includes a component that occurs primarily as the aircraft turns, as well as the gravitational acceleration. When estimating attitude, the accelerometer measurement terms other than gravitational ones can be considered as disturbances. Therefore, errors increase in accordance with the flight dynamics. The proposed algorithm is designed for using velocity as an aid for high accuracy at low cost. It effectively eliminates the disturbances of accelerometer measurements using the airspeed. The algorithm was verified using helicopter experimental data. The algorithm performance was confirmed through a comparison with an attitude estimate obtained from an attitude heading reference system based on a high accuracy optic gyro, which was employed as core attitude equipment in the helicopter.

## 1. Introduction

Attitude control is a fundamental component of a pilot’s control of a helicopter. The use of accurate attitude information is critical to that control. In the past, the mechanical gyroscope was commonly used in navigation equipment. Since then, the fiber optic gyroscope (FOG), or ring laser gyroscope (RLG), have become the most commonly used types of navigation equipment for attitude control. Recently, as the cost of the MEMS inertial sensors has decreased and its performance has improved, their range of utilization has broadened to include equipment used to estimate helicopter attitude. Algorithms for the use of the cost-effective MEMS inertial sensors are increasingly being developed. Many studies have employed the extended Kalman filter, which is often used in the aerospace field [1,2] and considers a linearized model. Nevertheless, its practical application is difficult on account of errors due to the model’s uncertainty and its lack of design intuitiveness [3,4,5].

On the other hand, a simpler type of complementary filter, which considers frequency characteristics, has been applied in various fields [6,7]. When designing the complementary filter for estimating attitude, the attitude value, which is estimated by the accelerometer, is applied with a low-pass filter to remove the high-frequency noise. The other attitude value, derived by the gyroscope output integration, is subjected to a high-pass filter to remove the low-frequency drift. This algorithm is called the all-pass type on account of the merging of the two different attitude values.

The accelerometer measures the respective translation and centrifugal acceleration, as well as the gravitational acceleration. When estimating the attitude using an accelerometer, any acceleration other than the gravitational acceleration acts as a disturbance. Consequently, errors of attitude derived by the accelerometer sometimes increase or decrease according to the flight dynamics. Many studies have thus analyzed both the dynamic characteristics of the application target and adapting the cutoff frequency of the complementary filter. To comprehend the dynamic characteristics, the accelerometer, gyroscope, and external sensor have been used [7,8]. Moreover, a look-up table or fuzzy logic was used as a method for choosing the optimum cutoff frequency in accordance with the dynamics [9,10,11,12]. Nevertheless, these methods can output unexpected results when unexpected dynamics are input. Because they are adapted to specific targets, their utilization is limited.

In this study, airspeed information was used. Thus, disturbance factors, other than the gravitational acceleration, were effectively removed. The subsequent disturbance-free acceleration and angular velocity were used to derive a robust attitude result, even under dynamic conditions, through the design of a simple complementary filter. The data used to verify the proposed algorithm were obtained by experimental flight tests of helicopter. The experiment employed Surion, a utility helicopter developed by Korea Aerospace Industries (KAI, Sacheon, Korea). The performance of the algorithm is confirmed by comparison with an attitude obtained from the attitude heading reference system (AHRS) based on high accuracy optic gyro, which served as the main instrument of the helicopter.

## 2. Attitude Estimation from Accelerometer

### 2.1. Conventional Method

The following equation dictates a vehicle’s equation of motion in terms of specific force, which are the accelerometers reading in body-fixed frame (BFF) [7]:

(1) $$\left[\begin{array}{c} f_{x}^{b}\\ f_{y}^{b}\\ f_{z}^{b} \end{array}\right]=\left[\begin{array}{c} {\dot{v}}_{x}^{b}\\ {\dot{v}}_{y}^{b}\\ {\dot{v}}_{z}^{b} \end{array}\right]+\left[-\begin{array}{ccc} 0 & v_{z}^{b} & -v_{y}^{b}\\ v_{z}^{b} & 0 & v_{x}^{b}\\ v_{y}^{b} & -v_{x}^{b} & 0 \end{array}\right]\left[\begin{array}{c} \omega_{x}^{b}\\ \omega_{y}^{b}\\ \omega_{z}^{b} \end{array}\right]-\left[\begin{array}{ccc} cos\psi cos\theta & sin\psi cos\theta & -sin\theta \\ -sin\psi cos\phi +cos\psi sin\theta sin\phi & cos\psi cos\phi +sin\psi sin\theta sin\phi & cos\theta sin\phi \\ sin\psi sin\phi +cos\psi sin\theta cos\phi & -cos\psi sin\phi +sin\psi sin\theta cos\phi & cos\theta cos\phi \end{array}\right]\left[\begin{array}{c} 0\\ 0\\ g \end{array}\right]+\eta$$

Equation (1) can be simply expressed as follows:

(2) $$f_{i}^{b}={\dot{v}}_{i}^{b}+\omega_{i}^{b}\times v_{i}^{b}-C_{n}^{b}g^{n},i\in x,y,z$$

where $f_{i}^{b}$ refers to the acceleration value measured by the accelerometer in BFF, ${\dot{v}}_{i}^{b}$ denotes the translational acceleration measured in BFF, $\omega_{i}^{b}$ represent the angular velocity in BFF, $v_{i}^{b}$ denotes velocity in BFF, $C_{n}^{b}$ refers to the coordinate transformation matrix from navigation frame to BFF, $g^{n}$ represents the gravitational acceleration in navigation frame, ϕ is roll angle, θ is pitch angle, ψ is yaw angle. and η is white noise. This noise can be ignored. When stationary or in a level flight condition (${\dot{v}}_{i}^{b},\omega_{i}^{b}\approx 0$), Equation (2) can be expressed as:

(3) $$f_{i}^{b}=-C_{n}^{b}g^{n},\;i\in x,y,z$$

(4) $$f_{x}^{b}=gsin\theta \;f_{y}^{b}=-gcos\theta sin\phi \;f_{z}^{b}=-gcos\theta cos\phi$$

(5) $$\phi_{acc}=Atan2\left(-f_{y}^{b},-f_{z}^{b}\right)$$

$$\theta_{acc}=Atan2\left(f_{x}^{b},\sqrt{f_{y}^{2}+f_{z}^{2}}\right)$$

In other words, the accelerometer can measure only the gravitational acceleration. When Equation (3) is rearranged by each axis, the result is as shown in Equation (4). Similarly, under stationary or level flight conditions, the accelerometer measurement can be used to derive the attitude (Equation (5)). However, under dynamic conditions, ${\dot{v}}_{i}^{b},\omega_{i}^{b}$ cannot be ignored. Therefore, Equation (5) becomes ineffective under the same conditions.

### 2.2. Improved Method; Estimation of Disturbance in Accelerometer Measurements

Considering helicopter dynamics, the acceleration components, which are actually measured, can be classified into three major parts: translational acceleration caused by acceleration and deceleration (${\dot{v}}_{i}^{b}$); acceleration caused by rotation ($\omega_{i}^{b}\times v_{i}^{b}$); and gravitational acceleration ($C_{n}^{b}g^{n}$). Although the velocity along the x-axis, measured within BFF, can be considered by using the helicopter pressure sensor, the translational accelerations and velocities along y and z axes can be ignored because it is assumed to very small $\left({\dot{v}}_{y}^{b},{\dot{v}}_{z}^{b},v_{y}^{b},v_{z}^{b}\approx 0\right)$. Air speed ($v_{x}^{b}$) is calculated as a function of the difference between total pressure and static pressure [13]. Airspeed be obtained from an air data computer (ADC) which has been widely used for avionic systems. When ${\dot{v}}_{y}^{b},{\dot{v}}_{z}^{b},v_{y}^{b},v_{z}^{b}\approx 0$, the acceleration measured (Equation (1)) can be arranged as:

(6) $$f_{x}^{b}={\dot{v}}_{x}^{b}+gsin\theta \;f_{y}^{b}=v_{x}^{b}\omega_{z}^{b}-gcos\theta sin\phi \;f_{z}^{b}=-v_{x}^{b}\omega_{y}^{b}-gcos\theta cos\phi$$

where $v_{x}^{b}$ refers to the velocity value measured by pressure sensor in BFF, ${\dot{v}}_{x}^{b}$ denotes the translational acceleration in BFF, and $\omega_{y}^{b}$ and $\omega_{z}^{b}$ represent the angular velocity in BFF. When estimating attitude, the terms accelerometer measurements (Equation (6)) other than gravitational one can be considered as ‘disturbance’. Thus, the ‘disturbance ($D_{i}^{b}$)’ in accelerometer measurements can be expressed as:

(7) $$D_{i}^{b}={\left[\begin{array}{ccc} {\dot{v}}_{x}^{b} & v_{x}^{b}\omega_{z}^{b}, & -v_{x}^{b}\omega_{y}^{b} \end{array}\right]}^{T}\;i\in x,y,z$$

$D_{i}^{b}$ can be expressed by the translational acceleration measured in BFF (${\dot{v}}_{i}^{b}$) and by the acceleration generated by the centrifugal force in BFF ($\omega_{i}^{b}\times v_{i}^{b}$). Based on the assumptions of Equation (8), the gyroscope airspeed and angular velocity can be used to estimate the disturbance ($D_{i}^{b}$). Moreover, the disturbance can be removed as:

(8) $${\hat{f}}_{i}^{b}=f_{i}^{b}-{\hat{D}}_{i}^{b},i\in x,y,z$$

where $f_{i}^{b}$ refers to the acceleration measured by the accelerometer in BFF, ${\hat{D}}_{i}^{b}$ is the disturbance acceleration estimated by using the airspeed and angular velocity in BFF, and ${\hat{f}}_{i}^{b}$ denotes the estimated acceleration that is free of disturbance. The airspeed and angular velocity of ${\hat{D}}_{i}^{b}$ can be estimated by using the measurements of the pressure sensor and gyroscope. By using the airspeed and angular velocity, the disturbances of the translational acceleration and acceleration by rotation are effectively removed. Because the corrected values of the accelerometer are devoid of disturbances, the values now only contain gravitational acceleration. Finally, attitude can be estimated from the estimated acceleration:

(9) $${\hat{\phi }}_{acc}=Atan2\left(-{\hat{f}}_{y}^{b}-{\hat{f}}_{z}^{b}\right)$$

$${\hat{\theta }}_{acc}=Atan2({\hat{f}}_{x}^{b},\sqrt{{\left({\hat{f}}_{y}^{b}\right)}^{2}+{\left({\hat{f}}_{z}^{b}\right)}^{2}})$$

Meanwhile, the translational acceleration (${\dot{v}}_{x}^{b}$) is not measured. However, ${\dot{v}}_{x}^{b}$ can be derived by differential of airspeed. When deriving ${\dot{v}}_{x}^{b}$, noise can be increased on account of the differential, which, in turn, increases errors. To estimate the translational acceleration (${\dot{v}}_{x}^{b}$), a Kalman filter with a simple form is used to design Equations (10) through Equation (12):

State Equation:

(10) x(k)=Ax(k−1)+w(k),w(k)~ N(0,1)

z(k)=Hx(k−1)+υ(k),υ(k)~ N(0,5)

State Variable:

(11) $$x\left(k\right)={\left[v_{x}^{b}\left(k\right)\;{\dot{v}}_{x}^{b}\left(k\right)\right]}^{T}$$

Model:

(12) $$A=\left[\begin{array}{cc} 1 & t\\ 0 & 1 \end{array}\right]\;t\;:Sampling\;Time,\;10\;\mathrm{ms}$$

H=[1 0]

where x(k) is the state vector containing the terms of interest for the system (airspeed ($v_{x}^{b}$) and acceleration (${\dot{v}}_{x}^{b}$)). A is the state transition matrix which applies the effect of each system state parameter at time k−1 on the system state at time k. w(k) is the vector containing the process noise terms for each parameter in the state vector. z(k) is the vector of measurement; in this paper $v_{x}^{b}\left(k\right)$. H is the transformation matrix that maps the state vector parameters into the measurement domain. ν(k) is the vector containing the measurement noise terms for each observation in the measurement vector. The measurement noise (w(k)) and system noise (ν(k)) are determined with consideration of the magnitude of the noise and its time lag by trial and error.

## 3. Improved Attitude Estimation Solution Scheme

A complementary filter for attitude estimation performs low-pass filtering on a low-frequency attitude estimate, obtained from accelerometer data, and high-pass filtering on a biased high-frequency attitude estimate, obtained by direct integration of gyroscope data, and fuses these estimates together to obtain an all-pass estimate of attitude. Although high frequency information of gyroscope is reasonably reliable, low frequency information, which induces drift, is not. On the other hand, the low frequency information of accelerometers is reasonably reliable, while their high frequency information, which induce high sensitivity noise and slow response, is not. Characterized by such adverse response features, the accelerometer and gyroscope can be applied with the all-pass-type complementary filter to enhance the performance. As mentioned above, the all-pass filter combines the low-pass filter and high-pass filter.

$$\dot{\Phi }=T\omega_{i}^{b},\Phi \in \phi ,\theta ,\psi$$

(13) $$\left[\begin{array}{c} \dot{\phi }\\ \dot{\theta }\\ \dot{\psi } \end{array}\right]=\left[\begin{array}{ccc} 1 & sin\phi tan\theta & cos\phi tan\theta \\ 0 & cos\phi & -sin\phi \\ 0 & sin\phi sec\theta & cos\phi sec\theta \end{array}\right]\left[\begin{array}{c} \omega_{x}^{b}\\ \omega_{y}^{b}\\ \omega_{z}^{b} \end{array}\right]$$

**Figure 1:**
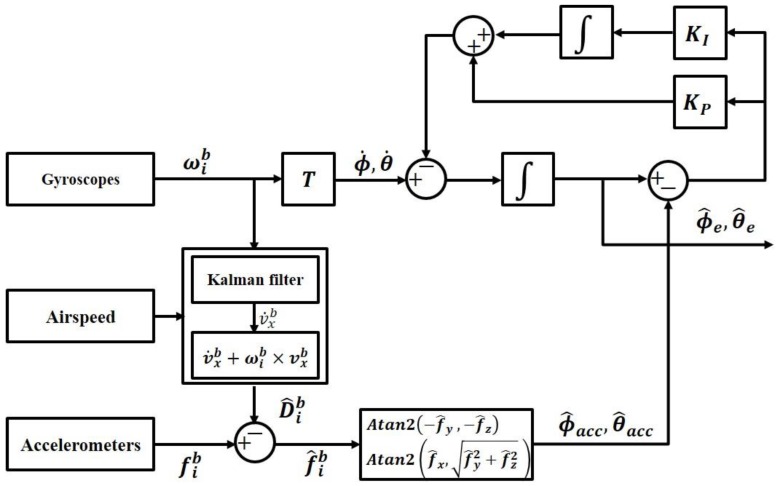
Airspeed-aided complementary filter.

Figure 1 presents a block diagram of the complementary filter using the measured airspeed, which enables robust attitude estimation, even in various dynamic situations. To this end, it employs the estimated acceleration $\left({\hat{f}}_{i}^{b}\right)$ that is devoid of disturbance acceleration $\left({\hat{D}}_{i}^{b}\right)$. Here, $\omega_{i}^{b}$ refers to the angular velocity measured by the gyroscope in BFF, and time rate of the Euler angles $\left(\dot{\phi },\dot{\theta }\right)$ can be obtained through transform matrix (T), as shown in Equation (13). In addition, $f_{i}^{b}$ represents the accelerometer-measured value in BFF, and, ${\hat{\phi }}_{acc}\;\mathrm{and}\;{\hat{\theta }}_{acc}$ are the attitude, which are estimated by using the acceleration value that is devoid of the disturbance acceleration. $K_{I}\;\mathrm{and}\;K_{P},$ respectively, refer to the integration gain and proportional gain of the filter. ${\hat{\phi }}_{e}\;\mathrm{and}\;{\hat{\theta }}_{e}$ are the consequential attitudes estimated by the airspeed-aided complementary filter. Figure 1 can be expressed in Laplace domain as follows:

(14) $${\hat{\Phi }}_{e}=\frac{1}{s}\dot{\Phi }+\frac{K_{P}}{s}\left({\hat{\Phi }}_{acc}-{\hat{\Phi }}_{e}\right)+\frac{K_{I}}{s^{2}}\left({\hat{\Phi }}_{acc}-{\hat{\Phi }}_{e}\right),\Phi \in \phi ,\theta$$

when Equation (15) is arranged from Equation (14), it can be respectively expressed by using the high-pass filter (HPF) part and low-pass filter (LPF) part:

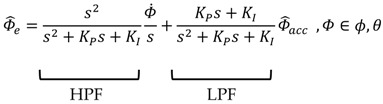
(15)


The integration gain ($K_{I}$) and proportional gain ($K_{P}$) are determined by setting the cutoff frequency ($\omega_{c}$) and damping ratio (ζ), as shown in Equation (16):

(16) $$K_{I}=\omega_{c}^{2},K_{P}=2\zeta \omega_{c}=\sqrt{2}\omega_{c},\;\left(when\;\zeta =0.707\right)$$

As shown in Equation (15), the complementary filter is a combination of the high-pass filter and low-pass filter, which share the same cutoff frequency ($\omega_{c}$) and are determined in accordance with the filter characteristics by $\omega_{c}$. By adjusting the weighted values of the two signals, the cutoff frequency can be appropriately configured to design the optimum complementary filter. Optimum cutoff frequency is determined experimentally by considering dynamic of helicopter aircraft. For the optimum algorithm, both gain are set as follows:

(17) $$K_{I}=0.015^{2},K_{P}=0.021\;\left(when\;\omega_{c}=0.015\right)$$

## 4. Experimental Verification

**Figure 2:**
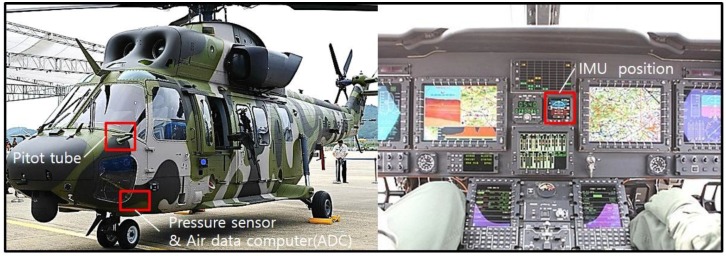
Korean utility helicopter, Surion.

Flight simulations were conducted to verify the helicopter attitude estimation algorithm using the measured airspeed, as suggested in this study. As mentioned earlier, the KAI Surion utility helicopter was used for the simulations (Figure 2). Table 1 and Table 2 summarize the basic specification of each sensor. The data used for the algorithm were obtained from the MEMS inertial sensor, which was applied as third-level equipment along with the Surion attitude instrument. The attitude estimation verification was conducted through a comparison of the above data and those obtained by AHRS based on a high accuracy optic gyro, the main instrument equipped on the helicopter.

**Figure 3:**
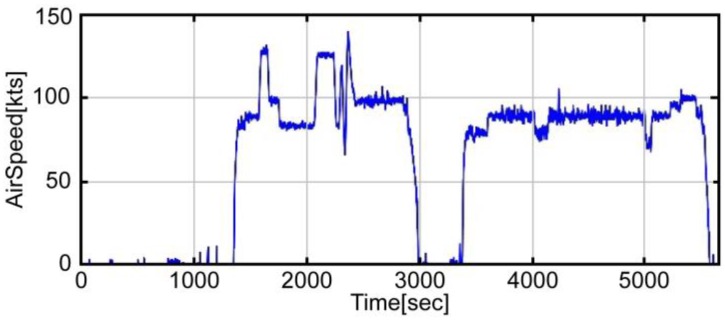
Airspeed.

**Figure 4:**
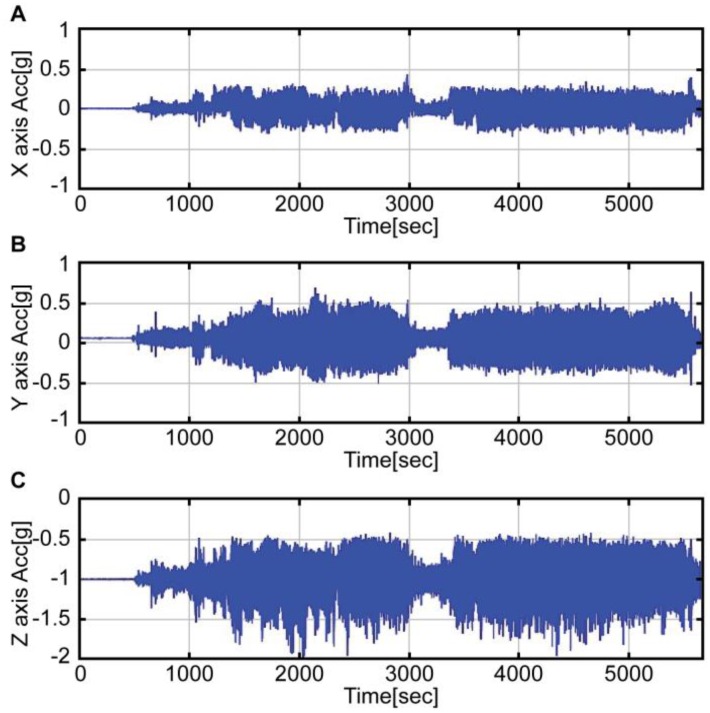
X, Y, Z acceleration by the MEMS accelerometer.

**Figure 5:**
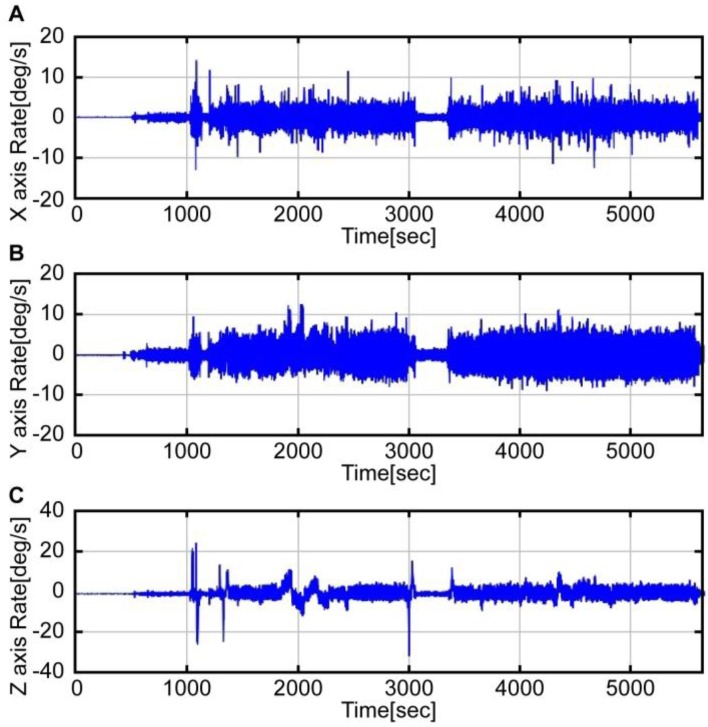
X, Y, Z rate by the MEMS gyroscope.

A flight test profile was conducted in accordance with an acceptance test procedure comprising a series of steps: take off, roll maneuver, pitch maneuver, landing, take off, loitering flight, and landing. Figure 3 shows the airspeed data obtained from the flight test, while Figure 4 and Figure 5 show the accelerometer data and gyroscope data obtained by the MEMS inertial sensor. As shown in the graphs of Figure 4 and Figure 5, large noises were introduced on account of the helicopter vibration.

Unlike the reference navigation equipment, which was mounted on the center of gravity (CG), the third-level navigation equipment was directly mounted on the instrument panel of the cockpit. It was thus exposed to more severe vibration conditions. Therefore, the filter had to be designed to remove the noise caused by the vibration. In this study, a Butterworth second-order low-pass filter was designed to appropriately remove the noise caused by the vibration.

**Figure 6:**
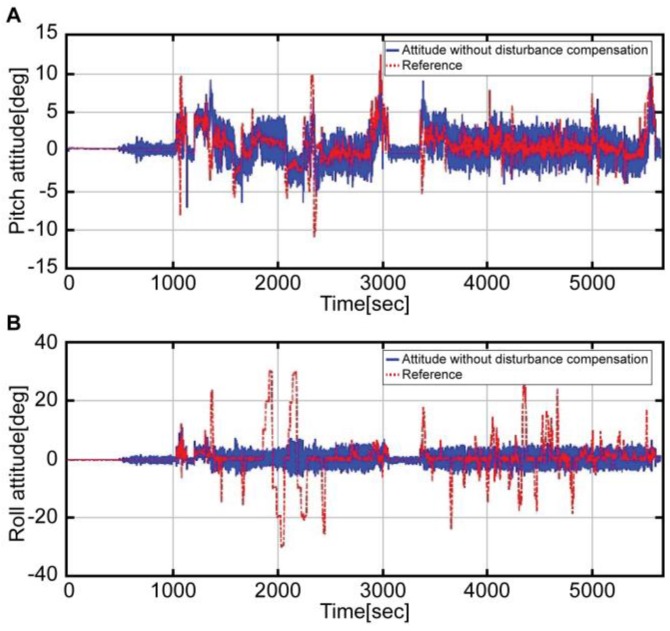
Attitude without compensation by the accelerometer (blue solid line) and Reference attitude (red dotted line); A: Pitch attitude, B: Roll attitude.

Figure 6 presents a graph comparing the attitude derived from Equation (5) (blue solid line) with the reference attitude (red dotted line). In both pitch (Figure 6A) and roll (Figure 6B) attitudes, the noise was more significant than in the reference attitude. In the case of the pitch attitude, although the noise was considerable, all the intervals, except the pitch maneuver interval, were similar to the reference. On the other hand, the roll attitude was not estimated for all intervals; it was only estimated for the respective intervals for landing and immediately after take-off (1000 s). Roll maneuver errors were usually greater than those of the pitch maneuver. This is because the disturbance acceleration was significant during the roll maneuver on account of the helicopter maneuver characteristics. Likewise, the attitude estimation, which used the raw data of the accelerometer, showed large errors during the dynamic flight maneuvers.

**Figure 7:**
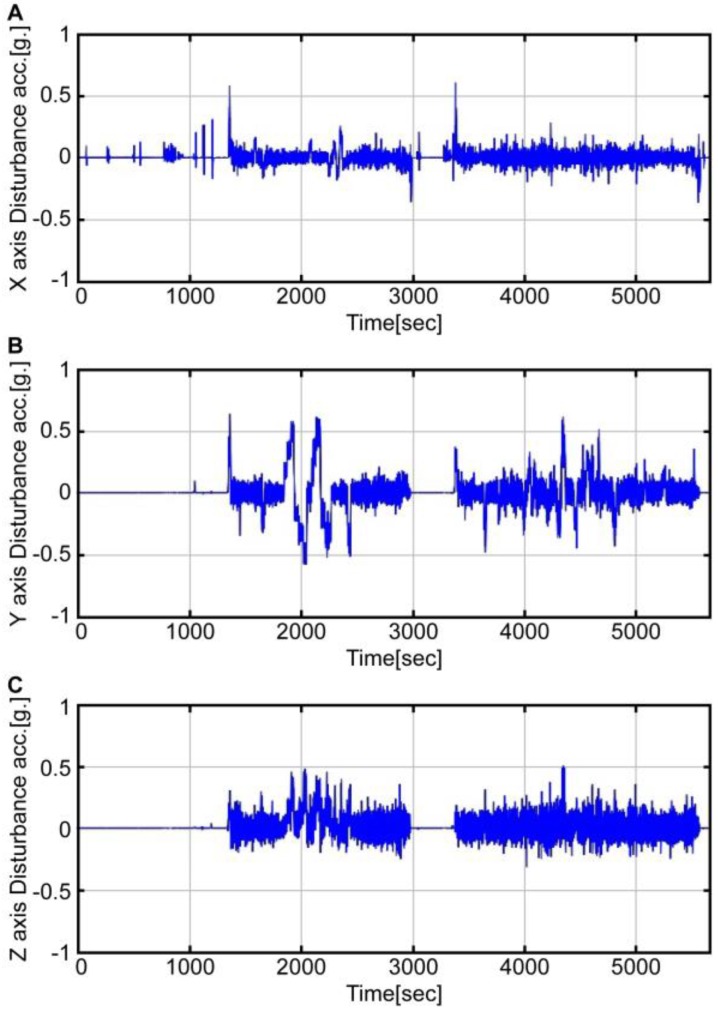
Disturbance acceleration on each axis.

Figure 7 presents a graph of disturbance acceleration along each axis. The disturbance acceleration along the x-axis is a translational acceleration estimated by using the airspeed ($v_{x}^{b}$), while the disturbance acceleration along y and z axes is a centrifugal acceleration estimated by using the airspeed and angular velocity ($\omega_{i}^{b}\times v_{i}^{b}$). As shown in the graph, during the roll maneuvering (1800 to 2300 s) and pitch maneuvering (2300 to 2400 s), significant disturbance acceleration is clearly visible. This conforms to the interval when errors occur in Figure 6. By removing the disturbance acceleration estimated from the accelerometer, the acceleration information can enable more accurate attitude estimation.

**Figure 8:**
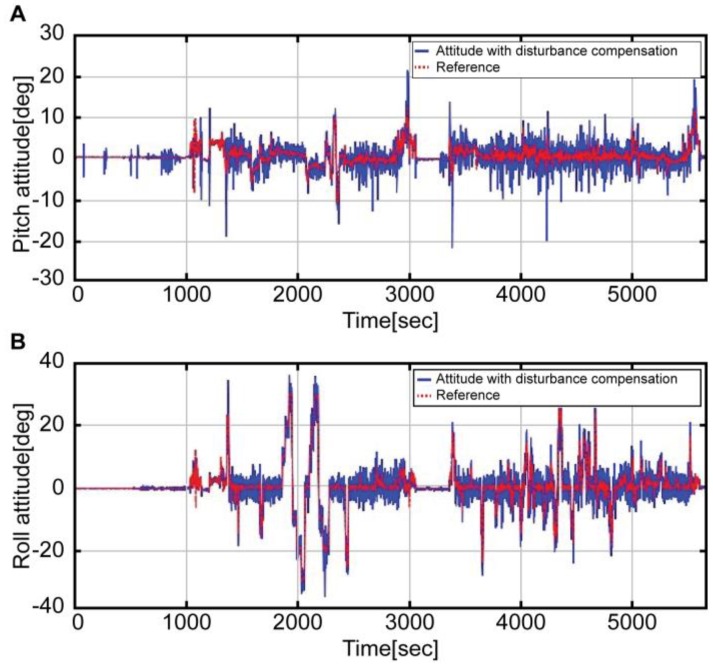
Attitude with compensation by the accelerometer (blue solid line) and Reference attitude (red dotted line); A: Pitch attitude, B: Roll attitude.

In Figure 8, the red dotted line refers to the reference attitude, while the blue solid line refers to the result obtained by removing the disturbance acceleration from the measured values of the accelerometer (Equation (9)).

**Figure 9:**
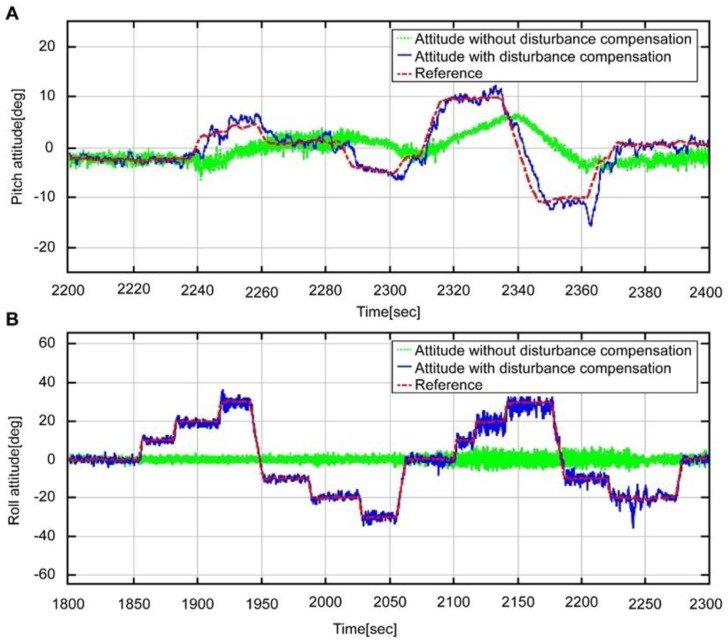
Attitude maneuver section by each accelerometer measurement and Reference attitude; A: Pitch attitude, B: Roll attitude.

The above result indicates that, unlike in Figure 6, the estimation is similar to that of the reference, regardless of the conditions. Meanwhile, Figure 9 shows the expanded interval graphs of pitch (Figure 9A) and roll (Figure 9B) attitudes measured by the accelerometer values (green dotted line), those with the disturbance removed (blue solid line), and the reference (red dotted line). Compared to the reference, significant noise and a time delay (≈1.5 s) exists; nevertheless, the blue line more accurately approximates the red line than the green line. The noise and time delay can be reduced by combining the angular velocity of the gyroscope.

**Figure 10:**
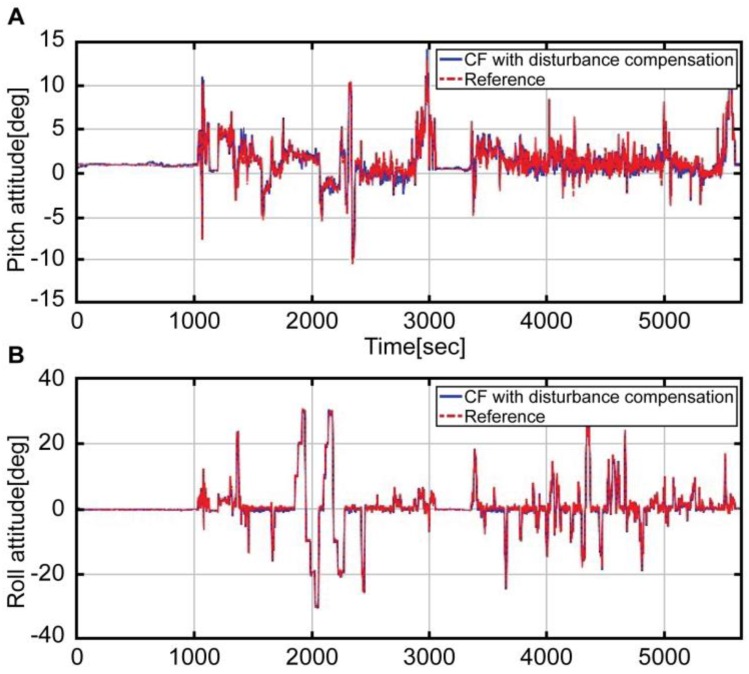
Attitude result with compensation by the complementary filter (blue solid line) and Reference attitude (red dotted line); A: Pitch attitude, B: Roll attitude.

**Figure 11:**
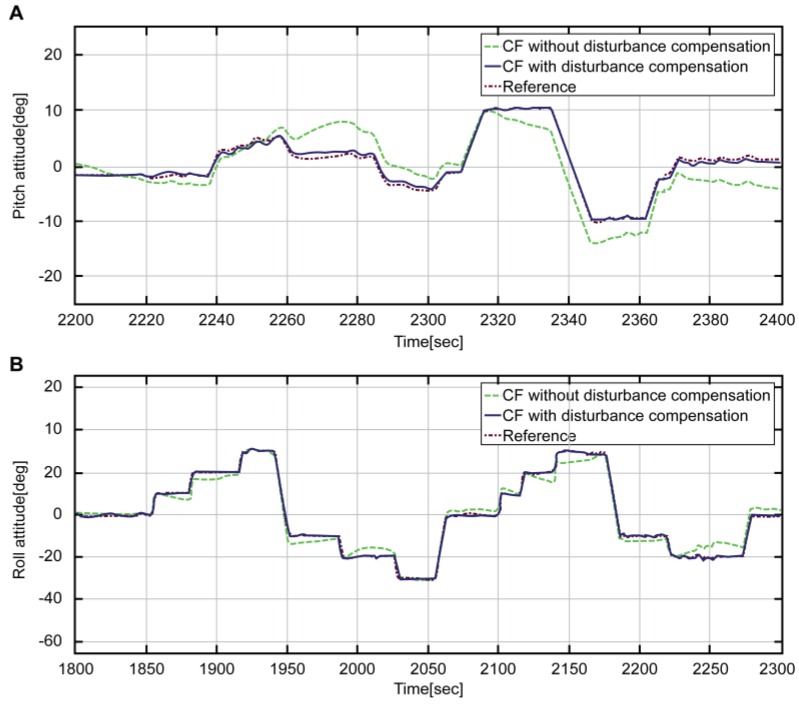
Attitude maneuver section by each complementary filter and Reference attitude; A: Pitch attitude, B: Roll attitude.

Figure 10 shows a graph comparing the attitude estimated by using the complementary filter with that of the reference. The blue solid line refers to the result obtained by fusing the estimated acceleration and the angular velocity devoid of disturbance through the complementary filter. The red solid line denotes the reference attitude. As shown in the graph, the reference of the entire interval is accurately estimated by the blue line. Moreover, Figure 11 refers to the expanded interval graph of the final estimated attitude, which compares the status before and after the correction of disturbance acceleration.

In Figure 11, the complementary filter designs are the same in both cases. The green dotted line refers to the attitude that is not corrected, while the blue solid line refers to the corrected attitude. The red dotted line refers to the reference attitude. It is evident that the blue line more effectively estimates the attitude. The green line, without consideration of disturbance, shows reduced errors through the merging with angular velocity; nevertheless, the errors still exist.

**Figure 12:**
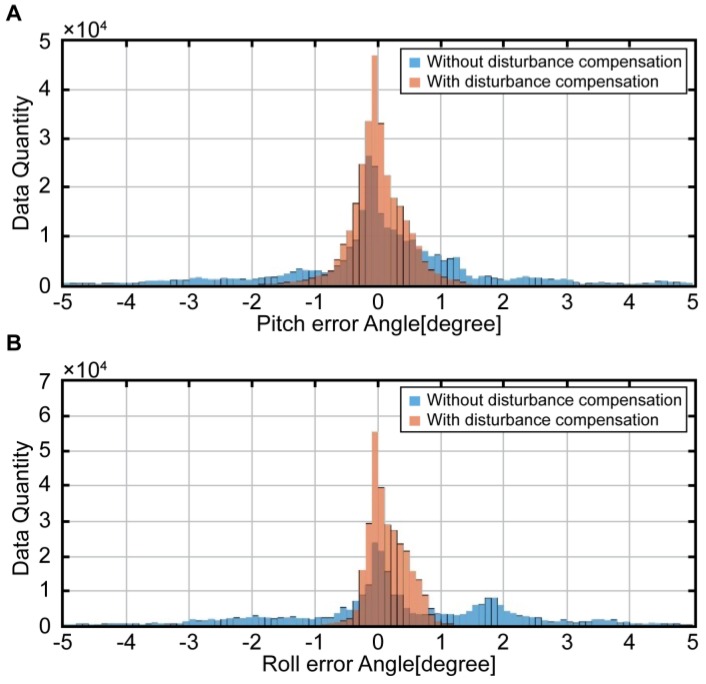
Histogram of attitude errors by each complementary filter; A: Pitch attitude error, B: Roll attitude error.

Figure 12 presents a histogram showing the errors between the reference attitude and estimated attitude. The blue histogram refers to the attitude errors of the complementary filter, in which the disturbance acceleration is not yet corrected. On the other hand, the red histogram shows the attitude errors of the complementary filter, in which the disturbance acceleration is corrected. The mean of the blue histogram is located on the center; therefore, no bias is detected. Nonetheless, the attitude errors are distributed widely among the entire intervals. In the red histogram, on the other hand, the errors are concentrated around zero. In other words, the complementary filter attitude errors, which are devoid of the disturbance acceleration, are significantly reduced, thus showing enhanced performance. In Table 3, these results are numerically compared.

In the table, $D_{i}^{b}=0$ refers to the result that does not consider the disturbance acceleration, whereas $D_{i}^{b}={\dot{v}}_{i}^{b}+\omega_{i}^{b}\times v_{i}^{b}$ refers to the case in which it is considered. The results of attitude estimation and errors of reference were compared using the root mean square (RMS) and standard deviation. The attitude errors of the complementary filter that considers disturbance acceleration show a reduction by approximately 75% and 83%, respectively, compared to the pitch attitude and roll attitude.

## 5. Conclusions

In this study, airspeed information was used and thus disturbance factors, other than the gravitational acceleration, were effectively removed from helicopter attitude estimations. Considering the helicopter environment, the disturbance acceleration equation was derived, and the disturbance acceleration was removed accordingly.

By designing a complementary filter that merges the acceleration that is devoid of disturbance with the gyroscope measurements, accurate estimations were effectively estimated, even under dynamic conditions. The experiment employed a Surion, a developed utility helicopter by Korea Aerospace Industries (KAI). The performance of the algorithm is confirmed by comparison with an attitude obtained from the attitude heading reference system (AHRS) based on high accuracy optic gyro, which serves as the main instrument of the helicopter.

## Figures and Tables

**Table 1:** IMU specifications (Analog devices ADIS 16488).

	Gyroscope	Accelerometer
Range	±450 °/s	±18 g
Resolution	0.02 °/s	0.8 mg
Nonlinearity	<0.01% of Full Scale	<0.1% of Full Scale
Bias stability	6.25 °/h,1σ	0.1 mg,1σ
Bandwidth	±0.2 °/s,1 σ	±14.8 mg,1 σ
Angle/Velocity Random Walk	330 Hz	330 Hz

**Table 2:** Pressure sensor specifications (Honeywell IPT).

	Pressure Sensor
Total Error	0.04% of Full Scale
Pressure Sensor Temperature Accuracy	±1.0 °C typical
Long Term Stability	0.025% of Full Scale max per year

**Table 3:** Attitude errors.

Condition	Attitude	RMS	Standard Deviation
$D_{i}^{b}=0$	Pitch	1.6498	1.6496
	Roll	1.8789	1.8677
$D_{i}^{b}={\dot{v}}_{i}^{b}+\omega_{i}^{b}\times v_{i}^{b}$	Pitch	0.4136	0.4136
	Roll	0.3371	0.3019
